# Supplementary material for: Pseudouridylation of 7SK by PUS7 regulates Pol II transcription elongation
Source: Nat Commun. 2025 Oct 30;16:9595. doi: 10.1038/s41467-025-64668-5 (PMC12575831; doi:10.1038/s41467-025-64668-5)
Supplement: Supplementary file 1 — Description of Additional Supplementary Files [file 41467_2025_64668_MOESM1_ESM.docx]

Description of Additional Supplementary Files

File name: Supplementary Data 1-4

Description: The processed data for BID-seq, PAR-CLIP, KAS-seq and mRNA-seq

File name: Supplementary Data 5

Description: primers and siRNAs used in this study
