# Supplementary material for: Pseudouridylation of 7SK by PUS7 regulates Pol II transcription elongation
Source: Nat Commun. 2025 Oct 30;16:9595. doi: 10.1038/s41467-025-64668-5 (PMC12575831; doi:10.1038/s41467-025-64668-5)
Supplement: Supplementary file 2 — Supplementary Information [file 41467_2025_64668_MOESM2_ESM.pdf]

Pseudouridylation of 7SK by PUS7 regulates Pol II transcription elongation

Yutao Zhao[1,2], Hui-Lung Sun[1,2], Wenlong Li[1,2], Chang Ye[1,2], Xiaoyang Dou[1,2], Yong Peng[1,2], Tong Wu[1,2], Pingluan Wang[1,2], Cheng-Wei Ju[1,2], Shun Liu[1,2], Yuhao Zhong[1,2], Qing Dai[1,2], Kinga Pajdzik[1,2], Chuan He[1,2,*]

[1]Department of Chemistry, Department of Biochemistry and Molecular Biology, and Institute for Biophysical Dynamics, The University of Chicago, Chicago, IL 60637, USA

[2]Howard Hughes Medical Institute, The University of Chicago, Chicago, IL 60637, USA

[*]Correspondence authors: Chuan He (chuanhe@uchicago.edu)

This file contains: **Supplementary Figure 1-9**

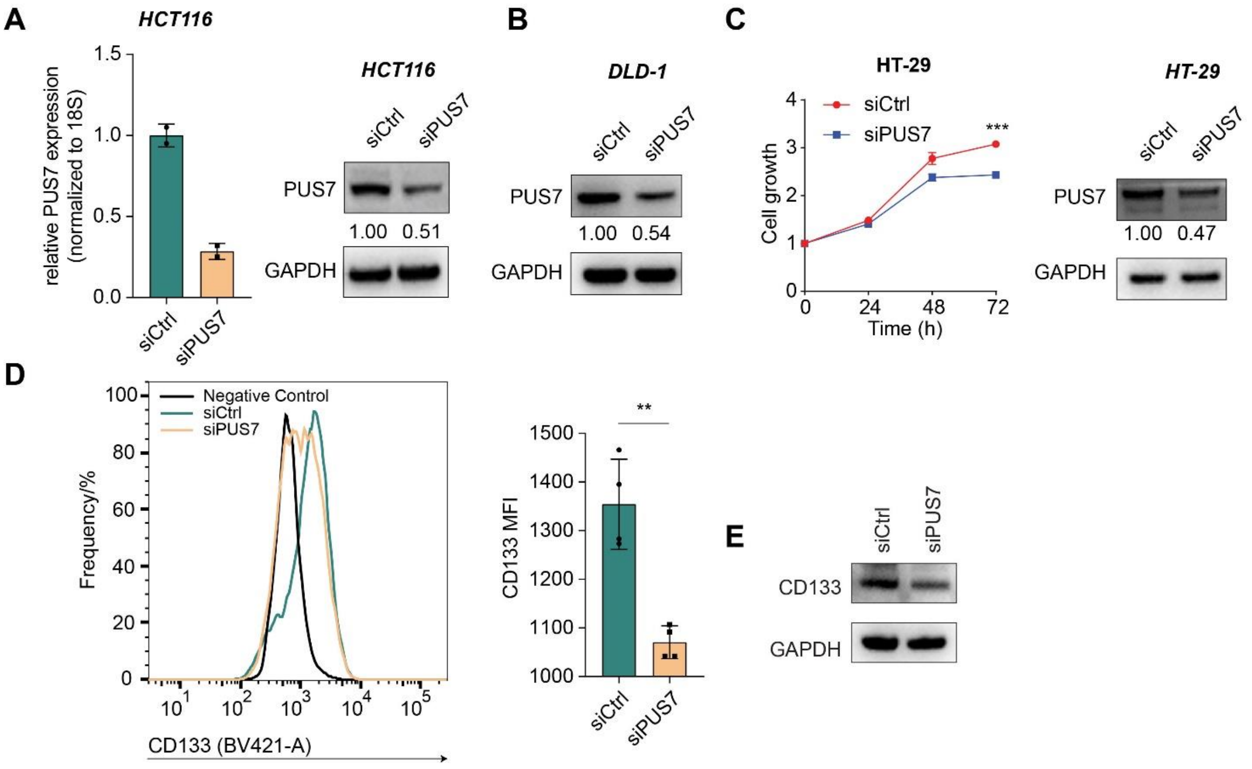

Supplementary Figure 1. ***PUS7* knockdown inhibits growth of colorectal cancer (CRC) cells and reduces stem cell population.** A) qPCR and western blot validating *PUS7* knockdown in HCT116 cells. qPCR data are presented as mean ± SEM (n = 2) and analyzed by a two-tailed Student's t-test. B) Western blot validating *PUS7* knockdown in DLD-1. C) Cell growth curve showing the effect of *PUS7* knockdown on HT-29 cells. Data are presented as mean ± SEM (n = 3) and analyzed by a two-tailed Student's t-test. Western blot validating *PUS7* knockdown in HT-29. D) Flow cytometry analysis showing the effect of *PUS7* knockdown on the stem cell population in HCT116 cells, with CD133 used as the stem cell marker. Bar plot quantifying the reduction in the stem cell population after *PUS7* knockdown. Data are presented as mean ± SEM (n = 4) and analyzed by a two-tailed Student's t-test. E) Western blot showing CD133 expression level with or without *PUS7* KD. p value significance: *p<0.05, **p<0.01, ***p<0.001, ****p<0.0001; ns, not significant. Source data are provided as a Source Data file.

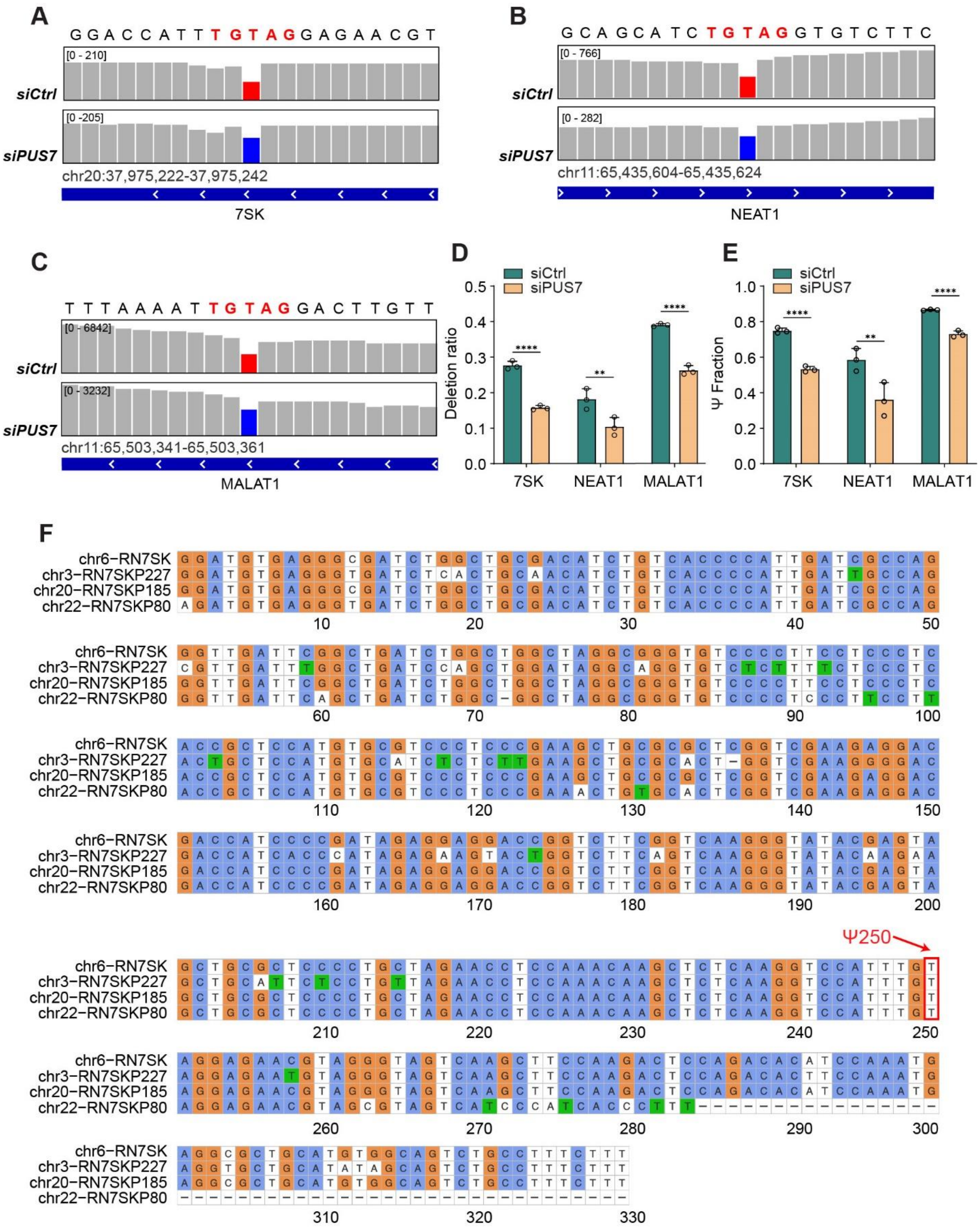

Supplementary Figure 2. ***PUS7* knockdown leads to hypo-pseudouridylation of 7SK, NEAT1 and MALAT1.** A) Read coverage plot showing the read coverage for each single base alongside the Ψ site on 7SK. B) Read coverage plot showing the read coverage for each single base alongside

the Ψ site on NEAT1. C) Read coverage plot showing the read coverage for each single base alongside the Ψ site on MALAT1. D) Bar plot showing the calculation of deletion ratio for 7SK, NEAT1, and MALAT1 in HCT116 cells with or without *PUS7* knockdown. Data are presented as mean ± SEM (n = 3), analyzed by two-tailed Student's t-test. E) Bar plot showing the calculation of Ψ fraction for 7SK, NEAT1, and MALAT1 in HCT116 cells with or without *PUS7* knockdown. Data are presented as mean ± SEM (n = 3), analyzed by two-tailed Student's t-test. F) Sequencing alignment of four 7SK transcripts (chr6:RN7SK, chr3:RN7SKP227, chr20:RN7SKP185 and chr22:RN7SKP80). The alignment was performed by R package "ggmsa". p value significance: *p<0.05, **p<0.01, ***p<0.001, ****p<0.0001; ns, not significant. Source data are provided as a Source Data file.

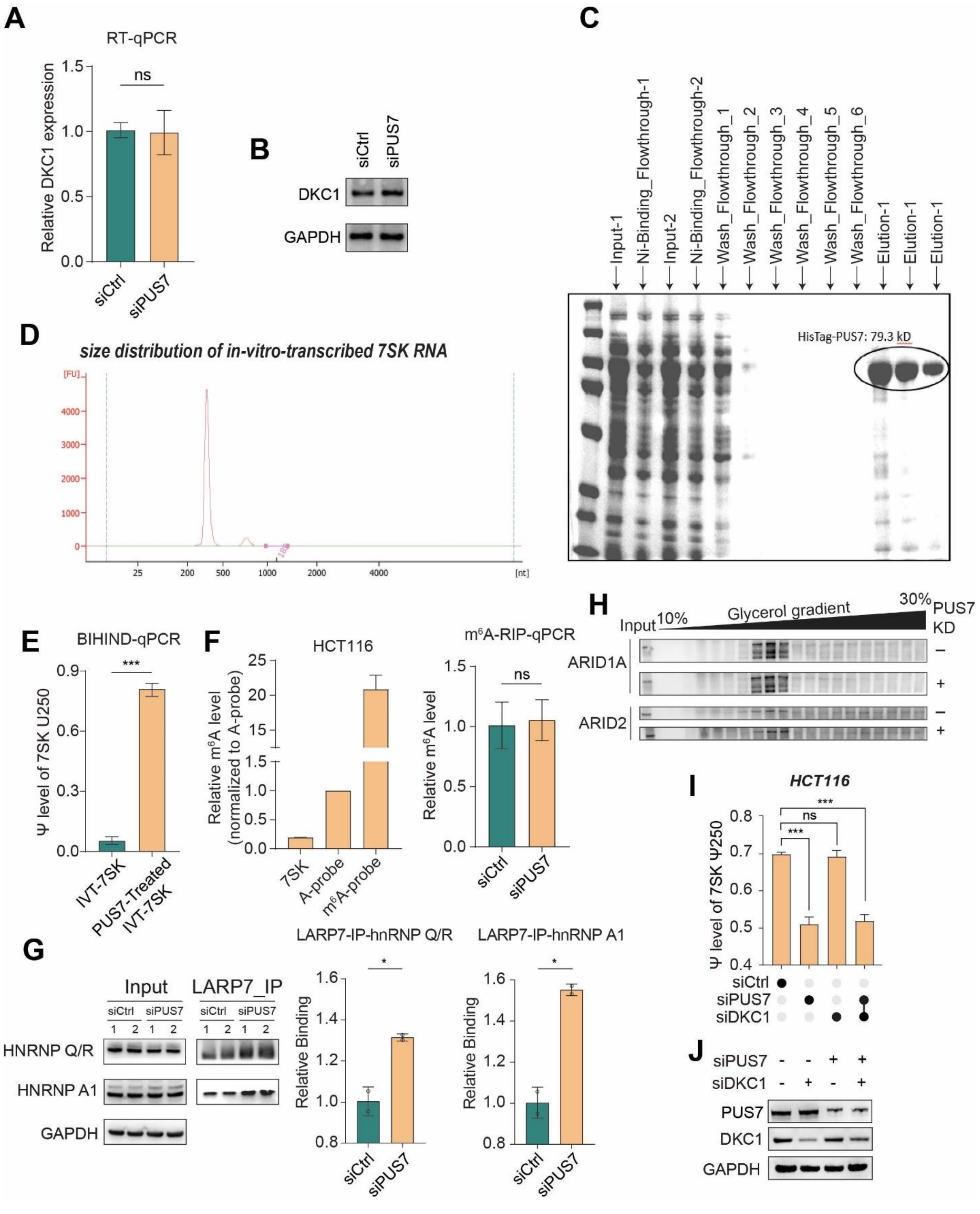

Supplementary Figure 3. **Identification of 7SK as one of PUS7's substrates.** A) RT-qPCR showing that *PUS7* knockdown does not affect RNA expression of DKC1 in HCT116 cells. B) Western blot showing that *PUS7* knockdown does not affect protein expression of DKC1 in

HCT116 cells. C) Expression and purification of His6-tagged PUS7 protein from *E. coli*. D) Size distribution of in-vitro-transcribed 7SK RNA. E) Bar plot showing Ψ level of PUS7-treated IVT-7SK. 64 pmol of PUS7 were incubated with 500 ng of in-vitro-transcribed 7SK at 37°C for 30 minutes. The recovered 7SK was subjected to BIHIND-qPCR for Ψ level measurement. F) Left: Bar plot showing the m6A level measurement based on m^6A-RIP-qPCR. The A-probe and m^6A-probe were derived from the EpiMark® N6-Methyladenosine Enrichment Kit (NEB). Right: m^6A-RIP-qPCR results showing no change on methylation levels on 7SK after *PUS7* knockdown. Data are shown as mean ± SEM (n = 2) and analyzed using two-tailed Student's t-tests. G) Western blot analysis of HNRNP Q/R and HNRNP A1 immunoprecipitated with antibodies against LARP7 with or without *PUS7* knockdown. Bar plot of quantification of HNRNP Q/R and HNRNP A1 levels co-immunoprecipitated with LARP7. Data are shown as mean ± SEM (n = 2) and analyzed using two-tailed Student's t-tests. H) Glycerol gradient sedimentation (10–30%) of HCT116 lysates followed by immunoblotting for ARID1A and ARID2, indicating that *PUS7* knockdown does not affect the size distribution of the BAF complex. I) Bar plot showing Ψ level of 7SK U250 with *PUS7* or *DKC1* knockdown. Data are shown as mean ± SEM (n = 3) and analyzed using two-tailed Student's t-tests. J) Western blot validating *PUS7* and *DKC1* knockdown in HCT116. p value significance: *p<0.05, **p<0.01, ***p<0.001, ****p<0.0001; ns, not significant. Source data are provided as a Source Data file.

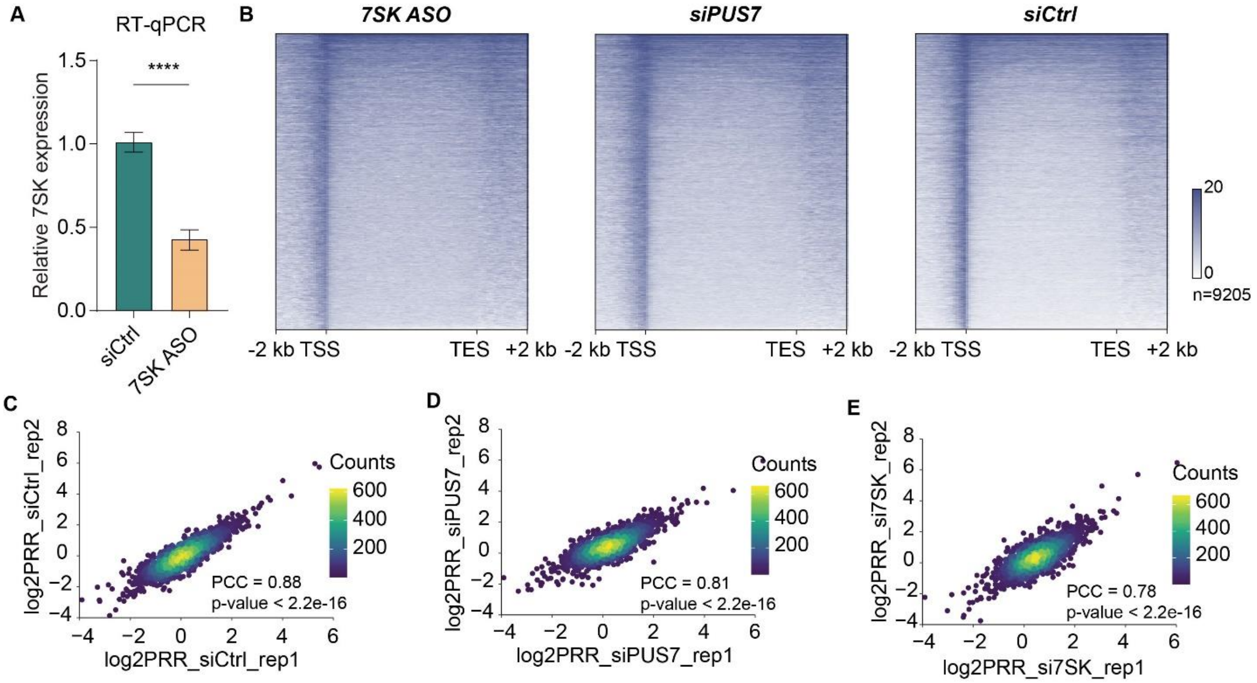

Supplementary Figure 4. **Quality control for KAS-seq in HCT116 cells with *PUS7* knockdown or 7SK depletion**. A) 7SK expression levels after 48 hours of 7SK ASO treatment. B) Heatmaps ranked by decreasing KAS-seq signals, showing increased Pol II occupancy in the gene body region after *PUS7* KD or 7SK ASO treatment. C) Correlation analyses of log2PRR values between replicates in the siCtrl group in HCT116 cells. D) Correlation analyses of log2PRR values between replicates in the siPUS7 group in HCT116 cells. E) Correlation analyses of log2PRR values between replicates in the 7SK ASO group in HCT116 cells. p value significance: *p<0.05, **p<0.01, ***p<0.001, ****p<0.0001; ns, not significant. Source data are provided as a Source Data file.

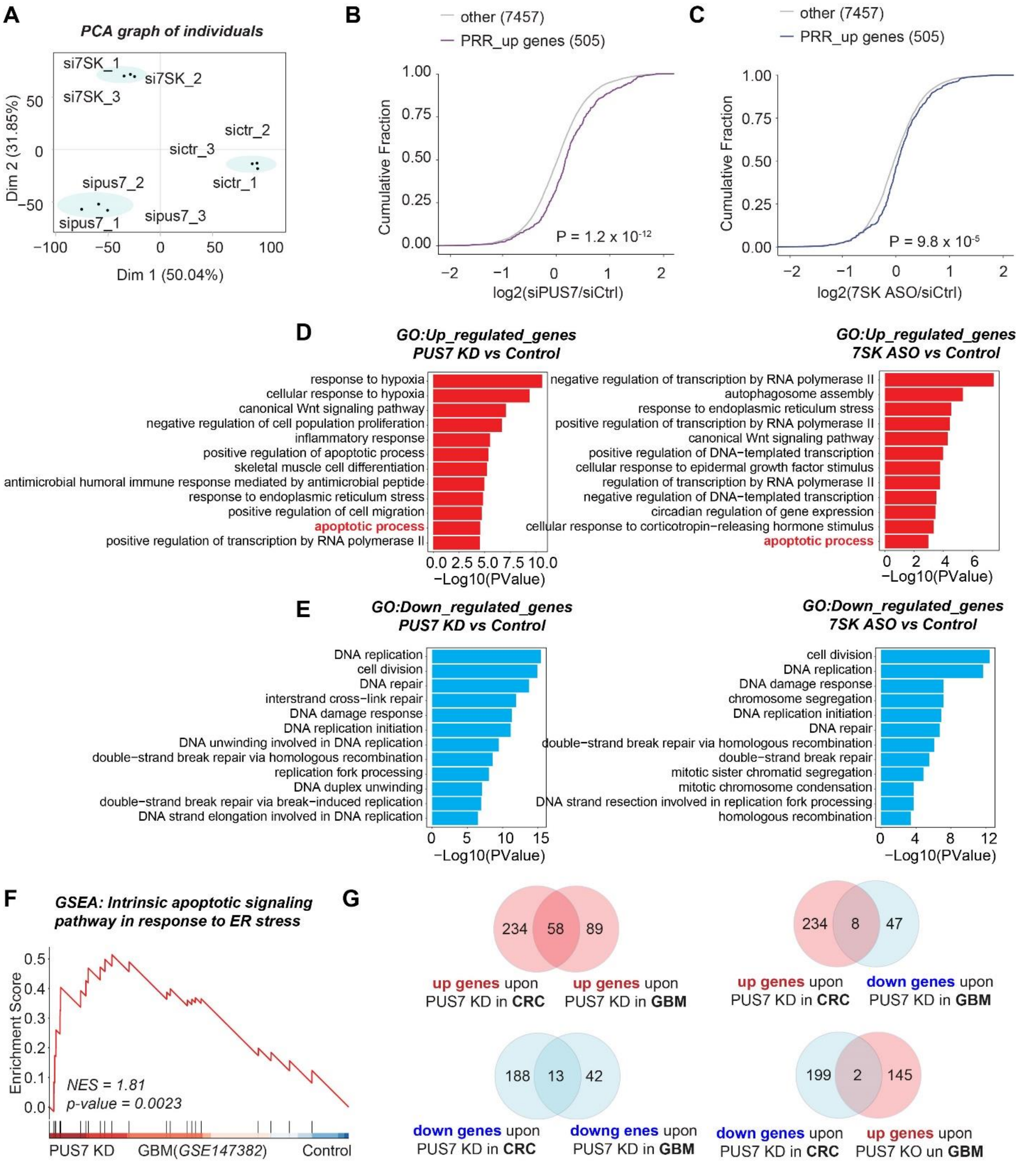

Supplementary Figure 5. **PUS7- and 7SK-deficient cells exhibit similar transcriptome alterations**. A) PCA plot of RNA-seq data, where each dot represents a sample. B) Cumulative distribution of gene expression changes upon *PUS7* knockdown, with the p-value determined using the Wilcoxon signed-rank test. C) Cumulative distribution of gene expression changes upon *7SK* knockdown, with the p-value determined using the Wilcoxon signed-rank test. D) GO analysis for upregulated genes in *PUS7* KD vs. control and 7SK ASO vs. control. E) GO analysis for

downregulated genes in *PUS7* KD vs. control and 7SK ASO vs. control. F) GSEA showing significant upregulation of the “intrinsic apoptotic signaling pathway in response to ER stress” in GBM stem cells upon *PUS7* KD (Dataset: GSE147382). G) Venn diagram illustrating the overlap of significantly altered transcripts between PUS7-depleted CRC cells and PUS7-depleted GBM stem cells (Dataset: GSE147382).

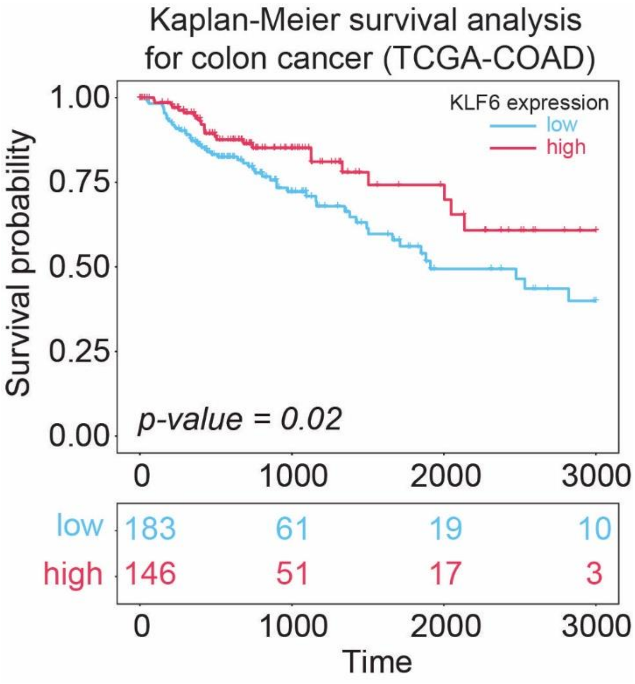

Supplementary Figure 6. **Kaplan-Meier analysis of the TCGA-COAD dataset showing that higher expression of KLF6 implies higher survival probability in colorectal cancer patients.** Source data are provided as a Source Data file.

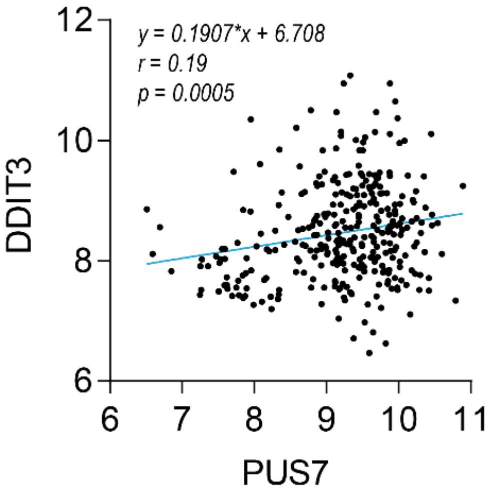

Supplementary Figure 7. **Correlation analysis between PUS7 and DDIT3 expression in the TCGA COAD dataset**. Pearson's correlation coefficient (r) is shown. Source data are provided as a Source Data file.

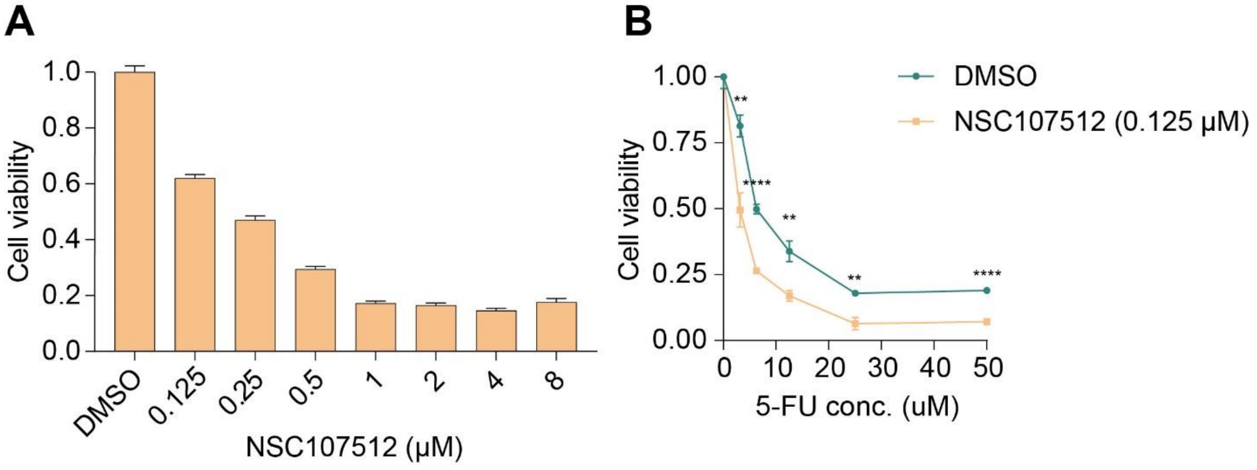

Supplementary Figure 8**. PUS7 inhibitor (NSC107512) sensitizes colon cancer cell HCT116 to 5-FU.** A) The cell viability of HCT116 with a series of concentrations of NSC107512. Data are shown as mean ± SEM (n = 3). B) Dose-response curves showing cell viability in HCT116 cells treated with 5-FU with or without NSC107512 (0.125 μM) treatment. Data are shown as mean ± SEM (n = 3) with two-tailed Student's t-tests. p value significance: *p<0.05, **p<0.01, ***p<0.001, ****p<0.0001; ns, not significant. Source data are provided as a Source Data file.

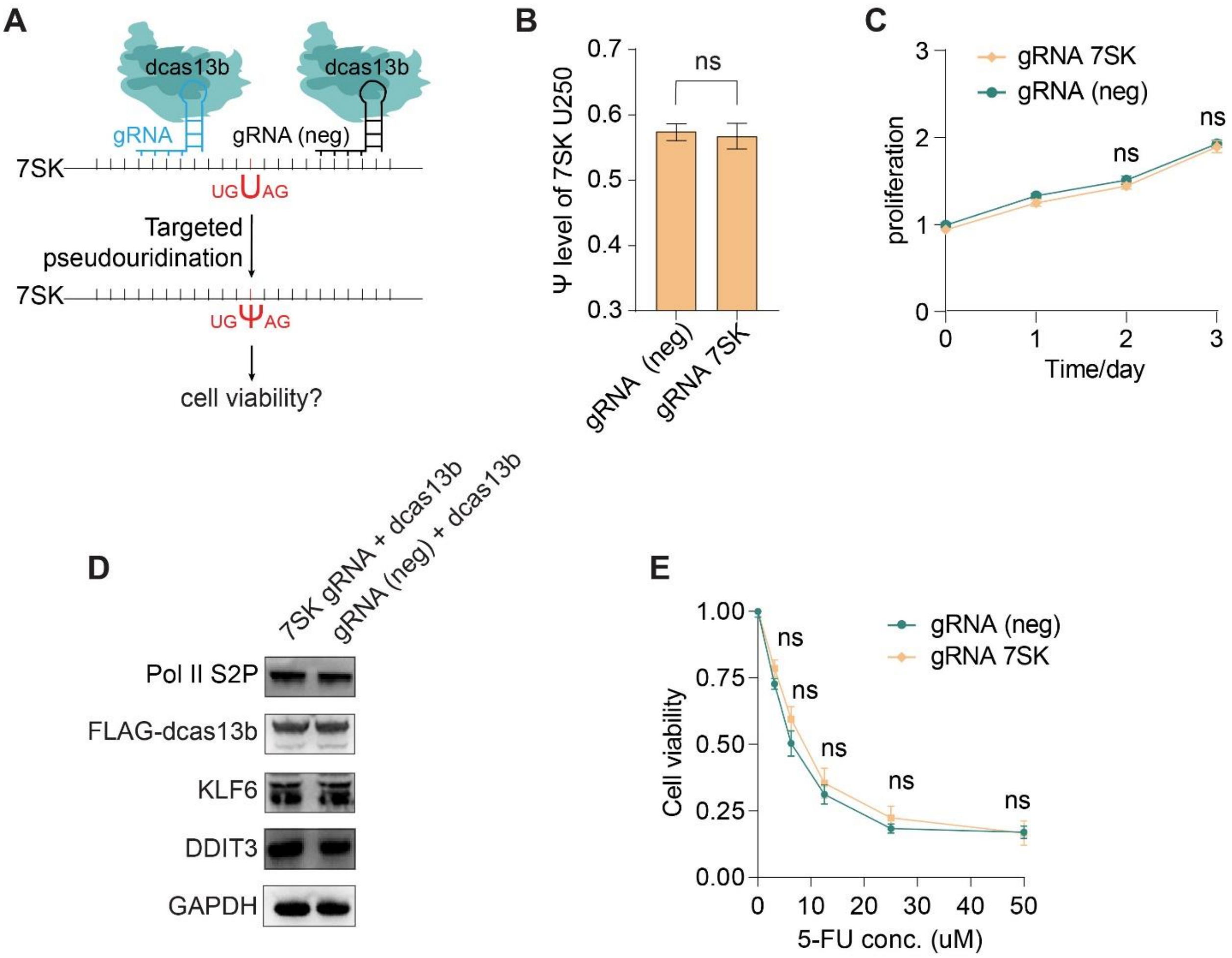

Supplementary Figure 9**. Validation of dCas13b-PUS7 specificity and impact on cellular processes.** A) Schematic of the dCas13b system. B) Bar plot showing the effects of dcas13b system on Ψ level of 7SK U250 with 7SK gRNA or negative gRNA. Ψ level was measured by BIHIND-qPCR. Data are shown as mean ± SEM (n = 3) with two-tailed Student's t-tests. C) Cell proliferation assay in HCT116 cells using the dCas13b system with 7SK gRNA or negative gRNA. Data are shown as mean ± SEM (n = 3) with two-tailed Student's t-tests. D) Western blot analysis of Pol II S2p, KLF6, and DDIT3 levels in HCT116 cells using the dCas13b system with 7SK gRNA or negative gRNA. E) Dose-response curves showing cell viability in HCT116 cells treated with 5-FU using the dCas13b system with 7SK gRNA or negative gRNA. Data are shown as mean ± SEM (n = 3) with two-tailed Student's t-tests. p value significance: $^*p<0.05$, $^{**}p<0.01$, $^{***}p<0.001$, $^{****}p<0.0001$; ns, not significant. Source data are provided as a Source Data file.
