# Supplementary material for: Pseudouridylation of 7SK by PUS7 regulates Pol II transcription elongation
Source: Nat Commun. 2025 Oct 30;16:9595. doi: 10.1038/s41467-025-64668-5 (PMC12575831; doi:10.1038/s41467-025-64668-5)
Supplement: Supplementary file 5 — Reporting Summary [file 41467_2025_64668_MOESM5_ESM.pdf]

nature portfolio | reporting summary

nature portfolio

Corresponding author(s): Chuan He

Last updated by author(s): Sep 10, 2025

Reporting Summary

Nature Portfolio wishes to improve the reproducibility of the work that we publish. This form provides structure for consistency and transparency in reporting. For further information on Nature Portfolio policies, see our Editorial Policies and the Editorial Policy Checklist.

Statistics

For all statistical analyses, confirm that the following items are present in the figure legend, table legend, main text, or Methods section.

n/a	Confirmed	
☐	☒	The exact sample size (*n*) for each experimental group/condition, given as a discrete number and unit of measurement
☐	☒	A statement on whether measurements were taken from distinct samples or whether the same sample was measured repeatedly
☐	☒	The statistical test(s) used AND whether they are one- or two-sided<br>*Only common tests should be described solely by name; describe more complex techniques in the Methods section.*
☒	☐	A description of all covariates tested
☒	☐	A description of any assumptions or corrections, such as tests of normality and adjustment for multiple comparisons
☐	☒	A full description of the statistical parameters including central tendency (e.g. means) or other basic estimates (e.g. regression coefficient) AND variation (e.g. standard deviation) or associated estimates of uncertainty (e.g. confidence intervals)
☐	☒	For null hypothesis testing, the test statistic (e.g. *F*, *t*, *r*) with confidence intervals, effect sizes, degrees of freedom and *P* value noted<br>*Give P values as exact values whenever suitable.*
☒	☐	For Bayesian analysis, information on the choice of priors and Markov chain Monte Carlo settings
☒	☐	For hierarchical and complex designs, identification of the appropriate level for tests and full reporting of outcomes
☐	☒	Estimates of effect sizes (e.g. Cohen's *d*, Pearson's *r*), indicating how they were calculated

Our web collection on statistics for biologists contains articles on many of the points above.

Software and code

Policy information about availability of computer code

Data collection	All libraries were sequenced on Illumina NovaSeqX
Data analysis	cutadapt tool (v4.8), BBMap tool (v.38.73), STAR mapping (2.7.11b), samtools (1.21), bowtie2 (2.4.4), PARalyzer (v1.5),deeptools (3.5.5), IGV (version 11.0.13), FeatureCounts (version 2.0.3)

For manuscripts utilizing custom algorithms or software that are central to the research but not yet described in published literature, software must be made available to editors and reviewers. We strongly encourage code deposition in a community repository (e.g. GitHub). See the Nature Portfolio guidelines for submitting code & software for further information.

Data

Policy information about availability of data

All manuscripts must include a data availability statement. This statement should provide the following information, where applicable:

- Accession codes, unique identifiers, or web links for publicly available datasets
- A description of any restrictions on data availability
- For clinical datasets or third party data, please ensure that the statement adheres to our policy

BID-seq, PAR-CLIP, KAS-seq and mRNA-seq data have been deposited at the Gene Expression Omnibus (GEO) under the accession number GEO: GSE288120.

Research involving human participants, their data, or biological material

Policy information about studies with human participants or human data. See also policy information about sex, gender (identity/presentation), and sexual orientation and race, ethnicity and racism.

Reporting on sex and gender	N/A. This work does not involve human research participants
Reporting on race, ethnicity, or other socially relevant groupings	N/A. This work does not involve human research participants
Population characteristics	N/A. This work does not involve human research participants
Recruitment	N/A. This work does not involve human research participants
Ethics oversight	N/A. This work does not involve human research participants

Note that full information on the approval of the study protocol must also be provided in the manuscript.

Field-specific reporting

Please select the one below that is the best fit for your research. If you are not sure, read the appropriate sections before making your selection.

☒ Life sciences ☐ Behavioural & social sciences ☐ Ecological, evolutionary & environmental sciences

For a reference copy of the document with all sections, see nature.com/documents/nr-reporting-summary-flat.pdf

Life sciences study design

All studies must disclose on these points even when the disclosure is negative.

Sample size	No statistical methods were used to predetermine sample size. Sample size were determined based on our prior experience on similar experiments and literature reports. For cell-based assays, samples were collected till we have sufficient number to obtain reliable statistics.
Data exclusions	No data were excluded.
Replication	Results were confirmed in at least two biological replicates for each experiment as stated in the figure legends. All attempts to replicate data are successful.
Randomization	The experiments were not randomized. Controlling for covariates was unnecessary because all assays were performed in pairs.
Blinding	Blinding was not applicable because the focus of this paper did not involve group allocation and blinding.

Reporting for specific materials, systems and methods

We require information from authors about some types of materials, experimental systems and methods used in many studies. Here, indicate whether each material, system or method listed is relevant to your study. If you are not sure if a list item applies to your research, read the appropriate section before selecting a response.

Materials & experimental systems

n/a	Involved in the study	
☐	☒	Antibodies
☐	☒	Eukaryotic cell lines
☒	☐	Palaeontology and archaeology
☒	☐	Animals and other organisms
☒	☐	Clinical data
☒	☐	Dual use research of concern
☒	☐	Plants

Methods

n/a	Involved in the study	
☒	☐	ChIP-seq
☐	☒	Flow cytometry
☒	☐	MRI-based neuroimaging

Antibodies

Antibodies used	The antibodies used in this study are listed below in the format of name (supplier, catalog): Rabbit anti-β-Tubulin (Cell Signaling Technology, 2128); Rabbit anti-SNRP70 (abcam, ab83306); Rabbit anti-H3 (Cell Signaling Technology, 9715); Rabbit anti-PUS7 for

PAR-CLIP and western blot (Bethyl Laboratories, A305-146A-T (thermo fisher)); Rabbit anti-GAPDH (Cell Signaling Technology, 2118); Rabbit anti-DKC1 (Cell Signaling Technology, 53234); Rabbit anti-LARP7 (Proteintech, 17067-1-AP); Rabbit anti-MEPCE (Proteintech, 14917-1-AP); Rabbit anti-HEXIM1 for immunoprecipitation (Cell Signaling Technology, 12604); Mouse anti-HEXIM1 for western blot (Proteintech, 66311-1-Ig); Rabbit anti-CDK9 (Proteintech, 11705-1-AP); Rabbit anti-ARID1A (Cell Signaling Technology, 12354); Rabbit anti-ARID2 (Cell Signaling Technology, 82342); Rabbit anti-CDK9 (Cell Signaling Technology, 53234); Mouse anti-Pol II (Biolegend, 664906); Rabbit Anti-KLF6 (Invitrogen, PA5-87561(thermo fisher)); Mouse anti-DDIT3 (Cell Signaling Technology, 2895); Goat anti-rabbit IgG-HRP (Cell Signaling Technology, 7074); Goat Anti-Mouse IgG H&L (Alexa Fluor® 488, abcam, ab150113); Goat Anti-Rabbit IgG H&L (Alexa Fluor® 594, abcam, ab150080); Horse anti-mouse IgG-HRP (Cell Signaling Technology, 7076).

Validation

Antibodies were validated by the manufacturers and used in accordance with the manufacturers recommendations

Eukaryotic cell lines

Policy information about cell lines and Sex and Gender in Research

Cell line source(s)

Human HCT116, DLD-1, HCT15 and HeLa cell lines used in this study were all purchased from ATCC (the American Type Culture Collection).

Authentication

Cell lines were not authenticated after purchase from ATCC.

Mycoplasma contamination

All cell lines used in this study were tested negative of mycoplasma contamination.

Commonly misidentified lines
(See ICLAC register)

No commonly misidentified line was used.

Plants

Seed stocks

N/A

Novel plant genotypes

N/A

Authentication

N/A

Flow Cytometry

Plots

Confirm that:

☒ The axis labels state the marker and fluorochrome used (e.g. CD4-FITC).

☒ The axis scales are clearly visible. Include numbers along axes only for bottom left plot of group (a 'group' is an analysis of identical markers).

☒ All plots are contour plots with outliers or pseudocolor plots.

☒ A numerical value for number of cells or percentage (with statistics) is provided.

Methodology

Sample preparation

HCT116 cells were trypsinized and washed with DPBS, then resuspended in Annexin V Binding Buffer (Biolegend) at a concentration of 1-5 x 106 cells/mL. To the cell suspension, 5 µL of FITC-conjugated Annexin V (Biolegend) was added to 100 µL of the cell suspension, followed by incubation for 15 minutes at room temperature, protected from light. After incubation, 2 mL Annexin V Binding Buffer was added, and the cells were centrifuged at 600 x g for 2 min at room temperature. The supernatant was discarded, and the cells were resuspended in 200 µL Annexin V Binding Buffer. To assess cell viability, 5 µL of Propidium Iodide Solution (Biolegend) was added, and the cells were incubated for 15 minutes at room temperature. The samples were analyzed by flow cytometry.

Instrument

BD LSR Fortessa flow cytometer

Software

FlowJo software (BD Biosciences) v10

Cell population abundance

About 50-60% cells were used in the final quantification. The gating of single cells excluded about 40% of the signals and the following gating for PI and FITC signals mostly included all the cells. Cells at different apoptotic stages were further quantified in FlowJo.

Gating strategy

Single cells gating (FSC-H vs A) followed by the gating confirmation with PI and FITC channels.

☒ Tick this box to confirm that a figure exemplifying the gating strategy is provided in the Supplementary Information.
